# TIR1-like auxin-receptors are involved in the regulation of plum fruit development

**DOI:** 10.1093/jxb/eru279

**Published:** 2014-07-04

**Authors:** Islam El-Sharkawy, Sherif M. Sherif, Brian Jones, Isabelle Mila, Prakash P. Kumar, Mondher Bouzayen, Subramanian Jayasankar

**Affiliations:** ^1^University of Guelph, Department of Plant Agriculture. 4890 Victoria Av. N., P.O. Box 7000 Vineland Station, ON, L0R 2E0 Canada; ^2^Faculty of Agriculture, Damanhour University, Damanhour, Egypt; ^3^The University of Sydney, Faculty of Agriculture, Australia; ^4^Université de Toulouse, INP-ENSA Toulouse, Génomique et Biotechnologie des Fruits, Castanet-Tolosan F-31326, France; ^5^Department of Biological Sciences, National University of Singapore, Singapore.

**Keywords:** Auxin-receptors, plum fruit development, *Prunus salicina*, subcellular localization, protein–protein interaction, auxin/ethylene crosstalk.

## Abstract

Ethylene has long been considered the key regulator of ripening in climacteric fruit. Recent evidence showed that auxin also plays an important role during fruit ripening, but the nature of the interaction between the two hormones has remained unclear. To understand the differences in ethylene- and auxin-related behaviours that might reveal how the two hormones interact, we compared two plum (*Prunus salicina* L.) cultivars with widely varying fruit development and ripening ontogeny. The early-ripening cultivar, Early Golden (EG), exhibited high endogenous auxin levels and auxin hypersensitivity during fruit development, while the late-ripening cultivar, V98041 (V9), displayed reduced auxin content and sensitivity. We show that exogenous auxin is capable of dramatically accelerating fruit development and ripening in plum, indicating that this hormone is actively involved in the ripening process. Further, we demonstrate that the variations in auxin sensitivity between plum cultivars could be partially due to *PslAFB5*, which encodes a *TIR1*-like auxin receptor. Two different *PslAFB5* alleles were identified, one (*Pslafb5*) inactive due to substitution of the conserved F-box amino acid residue Pro_61_ to Ser. The early-ripening cultivar, EG, exhibited homozygosity for the inactive allele; however, the late cultivar, V9, displayed a *PslAFB5*/*afb5* heterozygous genotype. Our results highlight the impact of auxin in stimulating fruit development, especially the ripening process and the potential for differential auxin sensitivity to alter important fruit developmental processes.

## Highlight text

A mutation in the plum auxin-receptor encoded by *PslAFB5*, which acts as a negative regulator of auxin responses, results in inactive Pslafb5 protein causing accelerated fruit-ontogeny events associated with auxin hypersensitivity.

## Introduction

Fruit and seeds are essential for human nutrition. Post-fertilization, fruits develop rapidly through a coordinated programme of molecular, biochemical and structural changes that optimize the potential for the development and dissemination of the seeds within. The fundamental importance of these processes has prompted considerable research into how they are governed. Research into fruit developmental processes has been greatly aided by analysing the outcome of both naturally occurring and induced genetic diversity (Kelly and Bradford, 1986; Lau *et al.*, 2008). One outcome of this research has been the identification of phytohormones as master regulators of the many processes involved (McAtee *et al.*, 2013). Ethylene, auxin, GA, ABA, and cytokinin have all been shown to play important integrative roles (Crane, 1969; Srivastava and Handa, 2005). Given its almost ubiquitous importance, it was not unexpected that auxin would play a prominent role in reproductive growth (Friml, 2003). Collectively, data from auxin application, quantification, and molecular genetic studies have shown that auxin is integral to most reproductive processes (Sedgley and Griffin, 1989; Sundberg and Østergaard, 2009). For example, auxin is required for floral meristem formation and acts with homeotic genes in determining floral organogenesis (Coen, 1992). Auxin is critical for the cell divisions that occur in response to fertilization (fruit-set), to the subsequent expansion and development of these cells (Mapelli *et al.*, 1978), and in regulating the onset and coordination of ripening processes (Gillaspy *et al.*, 1993; Trainotti *et al.*, 2007; El-Sharkawy *et al.*, 2010). Recent studies have shown that exogenous auxin accelerates fruit development and ethylene production, acting at least partially by triggering the expression of several genes, such as *ACS* and *ERF*, encoding ethylene-synthesis and -response components (Trainotti *et al.*, 2007; El-Sharkawy *et al.*, 2008, 2009).

Auxin responses are regulated at multiple levels, but they basically fall into two categories: responses that affect the hormone content and responses that regulate the response capacity (Lau *et al.*, 2008). Previous studies in tomato and *Arabidopsis* have identified auxin-signalling components, such as a member of the Aux/IAA family (*IAA9*) and two members of the Auxin Response Factor protein family, *ARF7* and *ARF8*, that are clearly important for early fruit development (Wang *et al.*, 2005; Goetz *et al.*, 2006, 2007; de Jong *et al.*, 2009*b*). Downregulation of these transcriptional regulators results in pleiotropic phenotypes, including fertilization-independent fruit-set.

The discovery that certain F-box proteins are auxin-receptors considerably improved the understanding of how auxin mediates cellular responses (Dharmasiri *et al.*, 2005*a*; Kepinski and Leyser, 2005). Basically, Aux/IAAs and ARF-transcriptional regulators interact in homo- and heterodimers, forming complexes that repress auxin-dependent changes in gene expression and therefore auxin action. Auxin binding to an F-box receptor promotes the binding of Aux/IAAs to the SCF^TIR1/AFB^ complex, leading to the ubiquitin-dependent proteolysis of the Aux/IAA (Dharmasiri and Estelle, 2004; Leiser, 2006). Loss of Aux/IAA repressors allows ARF-mediated auxin-responsive changes in gene transcription. Although this archetypal response mechanism is widespread, genetic studies have demonstrated alternative mechanisms. For example, TIR1- and AFB2-type auxin-receptors have been shown to be positive regulators of the auxin-response; however, loss-of-function studies revealed that members of the distinct AFB4 group act as negative regulators (Dharmasiri *et al.*, 2005*b*; Parry *et al.*, 2009; Greenham *et al.*, 2011). Given that auxin is a key regulator of early fruit development, it might be expected that *TIR1*-like receptors also regulate the process (Ren *et al.*, 2011).

In this study, we evaluated the role of auxin in flower and fruit development of two plum cultivars that vary in their flowering and ripening dates. We showed that the diversity in fruit development behaviours and auxin-responsiveness between the early-ripening/auxin-hypersensitive EG cultivar, and the late-ripening/reduced auxin-sensitivity V9 cultivar, could be partially due to a mutation occurred within the essential F-box domain of the auxin-receptor *PslAFB5*. This mutation resulted in a substitution in the highly conserved residue Pro_61_-to-Ser. Using yeast two-hybrid (Y2H) and bimolecular fluorescence complementation (BiFC) strategies we provided evidence that the allele with Pro_61_ (*PslAFB5*) encodes a functional auxin-receptor and the allele with the substitution Ser_61_ (*Pslafb5*) is inactive. Interestingly, genetic analysis of the two plum cultivars revealed that the early cultivar, EG, exhibited homozygosity for the inactive allele *gPslafb5*; however, the late cultivar, V9, displayed a *gPslAFB5*/*afb5* heterozygous genotype. The data presented here suggest that the auxin-hypersensitivity in the EG cultivar is at least partially due to the loss of PslAFB5 activity. Collectively, our results highlight the importance of auxin in coordinating the progression of fruit development, particularly in terms of the developmental phase transition that leads to the onset of ripening.

## Materials and methods

### Plant materials and post-harvest treatments

Reproductive structures from flower buds to ripening fruit were collected from two Japanese plum (*Prunus salicina* L.) cultivars, Early Golden (EG) and V98041 (V9). These were frozen in liquid-N_2_, and stored at –80°C until analysis. These two varieties were chosen according to the diversity of their responses to auxin application. Fruit were picked at stages S1 (first exponential growth phase), 16–34 and 32–50 days after bloom (DAB); S2 (pit hardening), 36–54 and 52–72 DAB; and S3 (second exponential growth phase), 57–77 and 74–108 DAB for EG and V9, respectively. Fruits from S4 (climacteric ripening) were collected from EG between 78 and 83 DAB, and from V9 between 110 and 128 DAB. To evaluate the effects of exogenous auxin in flower and fruit development, EG and V9 trees were sprayed during the reproductive cycle (flower bud and immature expanded fruit, stage 3) with two different concentrations of the synthetic auxin naphthalene acetic acid (NAA) (10 and 100 µM). Mature fruits of EG (76 DAB) and V9 (108 DAB) were harvested, surface sterilized, and subjected to various treatments, including: propylene (1000 µl l^–1^); the ethylene-inhibitor 1-MCP (1 µl l^–1^); NAA (10 and 100 µM for EG and V9, respectively); and the auxin transport-inhibitor TIBA (10 µM). Non-treated fruit were used as controls. See supplementary material for other details concerning hormone quantification, *PslTIR1/AFBs* sequence isolation and analysis, nucleic acid extraction, qPCR assays, and subcellular localization.

### Generation of truncated and mutated versions of PslTIR1-like proteins

Fragments containing N-terminal (∆N) and C-terminal (∆C) deletions of *PslTIR1*, *PslAFB2*, and *PslAFB5* were obtained by PCR using gene-specific primer pairs (primers 11–22, Supplementary Table S1 available at *JXB* online). Internal deletions of the F-box domain (∆F) from each sequence were created using two different strategies depending on the variation in N-terminal length (see supplementary material for further details). Mutants of *PslTIR1* (Pro_9_ to Ser) and *PslAFB5* (Pro_61_ to Ser) were created using the QuikChange site-directed mutagenesis kit (Stratagene, San Diego, CA, USA).

### Y2H assays

Yeast two-hybrid (Y2H) assays were performed using the Matchmaker Gold Yeast two-hybrid System (Clontech, Palo Alto, CA, USA). Full-length and modified *PslTIR1*, *PslAFB2*, and *PslAFB5* ORFs were cloned into the *Bam*HI-SalI site of the pGBKT7 bait vector (GAL4 binding-domain; DBD). Tomato and *Arabidopsis* cDNAs were fused into either the NdeI-BamHI site (*AtIAA7*) or the BglII-BamHI site (*ASK1*, *SlIAA3*, and *SlIAA9*) of the pGADT7 prey vector (GAL4 activation-domain; AD). Bait and prey vectors (100ng) were then introduced into Y2HGold and Y187 yeast strains, respectively, using Yeastmaker yeast transformation system 2. Interactions between the proteins were assayed by the mating method, according to the manufacturer’s instructions, in 96-well plates containing medium with or without 100 µM IAA. All assays were repeated at least three independent times.

### BiFC assays

Full-length and mutated *PslTIR1* and *PslAFB5* ORFs were fused into the SalI-BamHI site of the pSAT1-*N* vector, containing N-terminal YFP (NY). *ASK1* and *AtIAA7* sequences were cloned into the BglII-BamHI and XhoI-BamHI sites, respectively, of the pSAT1-*C* vector containing C-terminal YFP (CY). The different combinations of constructs encoding NY and CY at similar concentrations were mixed and then co-transfected into protoplasts obtained from suspension-cultured tobacco *BY-2* cells in the presence of 100 µM IAA, as previously described (El-Sharkawy *et al.*, 2014). Empty BiFC vectors (NY/CY), (NY:gene /CY) and (NY/CY:gene) were used as negative controls. All assays were repeated at least three times and visualized using confocal microscopy.

## Results

### Auxin enhances fruit development

**Fig. 1.**
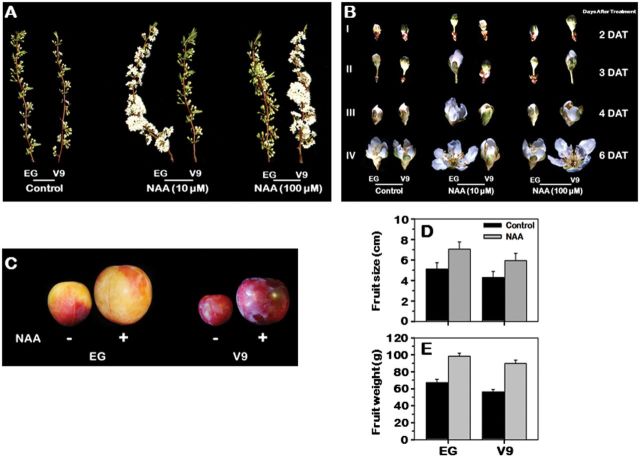
Variation in response to NAA application due to diversity in auxin sensitivity between early (EG) and late (V9) plum cultivars. In (A) and (B), the two plum cultivars were treated with two NAA concentrations (10 and 100 µM). Image (A) illustrates the effects of NAA concentration on flowering of EG and V9. Image (B) demonstrates the changes in flower developmental process due to auxin application. Floral stages were selected when ~70% of the flowers in the shoot were in the same developmental stage. I, II, III, and IV represent flower stages at 2, 3, 4, and 6 days after treatment (DAT). (C) Close-up views of plum fruits before and after auxin application, in which EG and V9 fruits were treated with 10 and 100 µM NAA, respectively. Images (D) and (E) show the alterations in EG and V9 fruit size and weight due to NAA treatment.

To assess the role of auxin in fruit development, a range of plum genotypes that vary in their flowering and ripening dates were selected and treated with NAA (Supplementary Table S2 available at *JXB* online). Two cultivars, early-ripening, EG, and late-ripening, V9, were selected for further analysis based on the diversity of their responses to the auxin treatment. Application of auxin to floral buds considerably enhanced flower development in both cultivars as determined by flower sizes at different developmental stages (Supplementary Table S3 available at *JXB* online). In EG, the maximum response to auxin in terms of flowering occurred at an NAA concentration of 10 µM. However, higher concentrations of NAA diminished responses in EG flowering (Fig. 1A, B; Supplementary Table S3 available at *JXB* online). In contrast, a flowering response to auxin application occurred only at a concentration of 100 µM in V9. In both cultivars, auxin treatment accelerated the fruit-set by 5±1 days compared to the untreated controls. Moreover, treatment of mature fruits from both cultivars with auxin resulted in significant increases in fruit size (~28%) and weight (~35%) over the untreated controls (Fig. 1C, D, E), indicating the potential for auxin to affect fruit development.

**Fig. 2.**
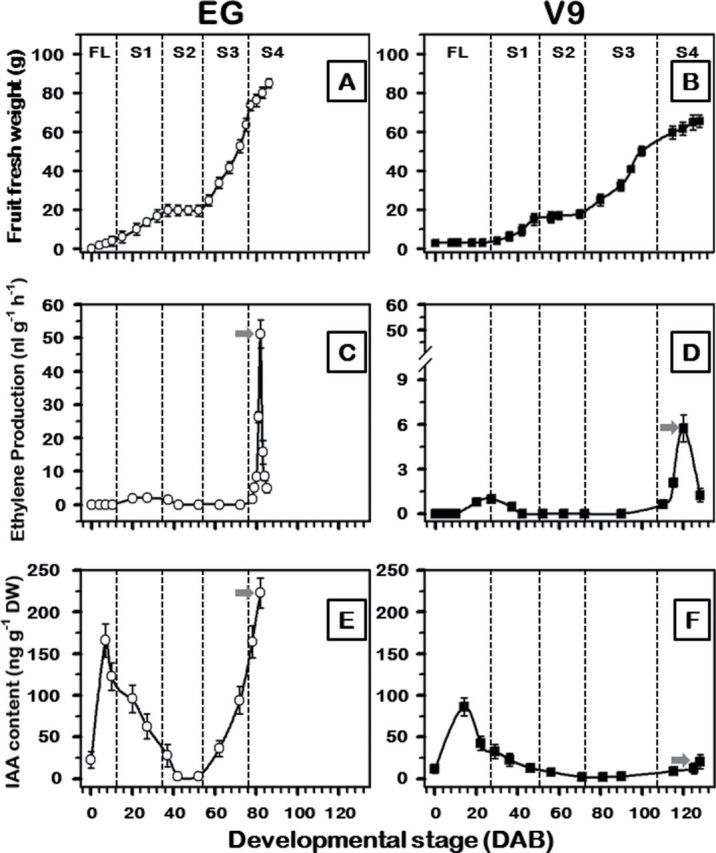
Representative fruit growth curves (A, B), changes in ethylene production (C, D), and IAA levels (E, F) during plum fruit development for the early- and late-ripening cultivars EG and V9, respectively. Values represent the mean ±SD, as derived from nine independent replicates (5 fruit per replicate). The x axis represents the developmental stages indicated by DAB. The developmental stages include flower (FL), and stages 1–4 (S1–S4) of fruit development. The y axis refers to the changes in fruit fresh mass (A, B), ethylene levels (C, D) and IAA content (E, F) during development, respectively. The grey arrow (C–F) represents the fruit at climacteric stage.

Ripening is the final stage of fruit development. Many of the processes integral to the ripening of plums and other climacteric fruit are known to be triggered by ethylene (Lelièvre *et al.*, 1997). To examine the contribution of auxin to the coordination of fruit development, the onset of ethylene production, and ripening, we monitored the ethylene production and endogenous IAA levels during EG and V9 fruit development. In stone fruit, including plum, the fruit growth followed a typical double-sigmoidal curve (Fig. 2A, B), in which four developmental stages (S1–S4) can clearly be recognized (El-Sharkawy *et al.*, 2007). V9 exhibited significant delays in fruit developmental progression compared to EG, due to a delay in flowering (16±2 days later than in EG) and extended S3 duration (14 d± 2 days longer than in EG), resulting in prolonged fruiting period (Supplementary Table S2 available at *JXB* online). Ethylene was undetectable in immature fruit, but both cultivars produced significant amounts of ethylene during ripening (Fig. 2C, D). EG fruit, in particular exhibited a sharp increase in ethylene production during ripening. In contrast, the onset of ripening was delayed in V9 (22±2 days later than in EG) with lower ethylene levels, resulting in slower ripening process (13±2 days longer than in EG).

The IAA accumulation patterns were similar in the two cultivars during the early stages of fruit development (Fig. 2E, F). Endogenous IAA levels were low at anthesis, but rose rapidly in flowers post-fertilization. IAA levels gradually declined thereafter in both cultivars to low or undetectable levels by S2. A clear divergence in IAA accumulation profiles between the two cultivars occurred subsequent to this minimum. In EG, the concentration of endogenous IAA increased rapidly as the fruit matured, reaching maximal levels during climacteric ripening. In stark contrast, the levels of IAA increased only slightly in V9, and remained low through to the end of the ripening phase.

Auxin application accelerated the onset of ripening; thus, to ascertain the relationship between the auxin treatment and ethylene production and ripening, ethylene was assessed during the ripening of EG and V9 fruits pre-exposed to different treatments designed to alter auxin- or ethylene-responses. EG and V9 fruit displayed a climacteric peak of ethylene production at ~5 days and ~13 days after the onset of ethylene production, respectively. Ethylene emission at the peak of the climacteric was 48.2±4.2 and 5.7±0.9 nl g^–1^ h^–1^ for EG and V9, respectively. Propylene-treatment accelerated ripening and increased ethylene production in both cultivars. Maximal levels of ethylene were observed at ~2 days (EG) and ~5 days (V9) post-treatment with a correspondingly increased ethylene production of 80.5±5.1 and 58.6±3.2 nl g^–1^ h^–1^ for EG and V9, respectively (Supplementary Figure S1A, B available at *JXB* online). As expected, all fruit treated with the ethylene response inhibitor, 1-MCP, were unable to ripen autonomously and their ethylene production remained low (Supplementary Figure S1C, D available at *JXB* online).

Auxin dramatically accelerated the ripening of both plum cultivars, but in a slightly different manner for each one. Ethylene production in the auxin-treated EG fruit peaked at a high level (69.5±5.5 nl g^–1^ h^–1^) ~3 days after treatment. Relative to NAA-treated EG fruit, a lower climacteric ethylene peak of 30.6±2.2 nl g^–1^ h^–1^ was observed in NAA-treated V9 fruit and this peak occurred much later, ~15 days after the treatment (Supplementary Figure S1E, F available at *JXB* online). Treatment with the auxin transport inhibitor TIBA had almost no effect on ethylene production in EG, but had more pronounced effects in V9 (Supplementary Figure S1G, H available at *JXB* online), as determined by the time necessary to have the climacteric peak after the treatment and the levels of ethylene at the peak.

### Identification and characterization of *PslTIR1/AFB* genes

To investigate the molecular basis of auxin action in fruit development, genes encoding three novel proteins closely related to the F-box *TIR1*-like gene family of auxin-receptors were isolated from plum. The isolated sequences were designated as *PslTIR1*, *PslAFB2*, and *PslAFB5* to comply with the names retained for the homologues from other plant species. The relationships between the predicted plum and *Arabidopsis* amino acid sequences (Dharmasiri *et al.*, 2005*b*; Walsh *et al.*, 2006), as indicated by percentage similarity over the whole sequence, are presented in Supplementary Table S4 available at *JXB* online. Despite strong sequence diversity among the different gene members, there were also highly homologous sequences putatively coding for closely related proteins. Strong sequence similarity between specific plum and *Arabidopsis* sequences (80–89%) indicates that these sequences are likely to be orthologous. The derived *PslTIR1/AFB* sequences comprise three typical domains that are associated with the F-box subunit protein of SCF (E3) ubiquitin ligase (Supplementary Figure S2 available at *JXB* online). The deduced amino acid sequences of *PslTIR1* and *PslAFBs* start by an N-terminal domain with unknown function that exhibits high sequence divergence in length [3–58 amino acids (aa)] and homology (12–66% similarity), followed by the characteristic F-box domain (42–48 aa), which shows higher sequence similarity, ranging from 65–81%. Finally, the C-terminal domain, which is composed of several LRRs, occupies the major part of the predicted protein (521–535 aa; 64–90% similarity). The number of LRRs varied among the different sequences [predicted to be 5- (*PslAFB5*), 6- (*PslTIR1*), and 7-LRRs (*PslAFB2*)]. See supplementary material for gene structure and organization.

### PslTIR1/AFBs are components of an SCF complex that interacts with Aux/IAAs

**Fig. 3.**
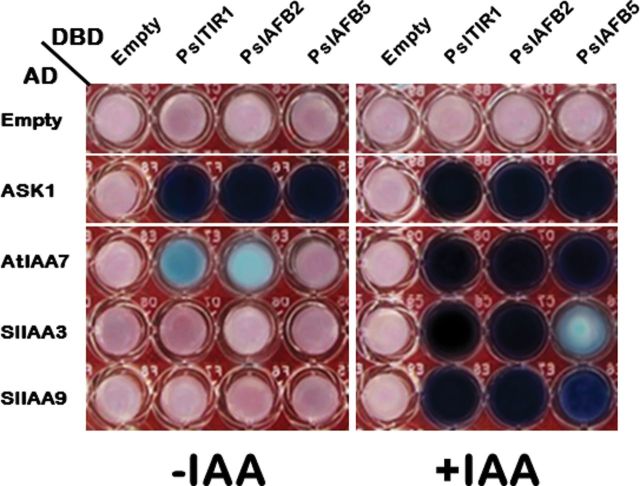
Differences in PslTIR1/AFBs–ASK1 and –Aux/IAAs interactions in yeast. Y2H assays of the interaction (indicated by formation of blue colour) were performed using PslTIR1/AFBs (as bait) in Y2HGold strain yeast and ASK1 or Aux/IAAs (as prey) in Y187 strain yeast. The mated yeast (DBD–PslTIR1/AFBs and AD–ASK1 or –Aux/IAAs) were grown in 96-well plates containing DDO/X/A selective medium in the presence or absence of 100 µM IAA. All experiments were repeated at least three times.

F-box subunits are anchored into SCF complexes via binding of the F-box domain to Skp1 (Zheng *et al.*, 2002). If PslTIR1/AFB proteins are part of a functional SCF^PslTIR1/AFBs^ complex, they should interact with one or more Skp1 homologues (Gray *et al.*, 1999). We tested for the capacity of PslTIR1/AFBs to interact with the *Arabidopsis* Skp1-related protein (ASK1) using Y2H. Consistent with the *Arabidopsis* TIR1-like proteins, all PslTIR1/AFBs interacted strongly with ASK1 in a manner independent of auxin action (Fig. 3). We next sought to determine if Aux/IAA proteins are potential substrates of the SCF^PslTIR1/AFBs^ E3 ubiquitin–ligase by assessing interactions between PslTIR1/AFBs and several Aux/IAAs using the Y2H system in the presence or absence of IAA (Fig. 3). The well-characterized Aux/IAA proteins chosen for this study were the *Arabidopsis* IAA7 (AtIAA7), and the tomato IAA3 and IAA9 (SlIAA3 and SlIAA9), which represent three different sub-clades of Aux/IAAs and play distinct roles in mediating auxin responses *in planta* (Nagpal *et al.*, 2000; Wang *et al.*, 2005; Chaabouni *et al.*, 2009). The analysis revealed a clear difference in the binding specificity and auxin-dependence of the PslTIR1/AFB proteins for the various Aux/IAAs. The binding results confirmed the auxin-induced assembly of stable IAA::PslTIR1/AFBs::Aux/IAAs co-receptors in yeast.

### Characterization of PslTIR1/AFBs domains

**Fig. 4.**
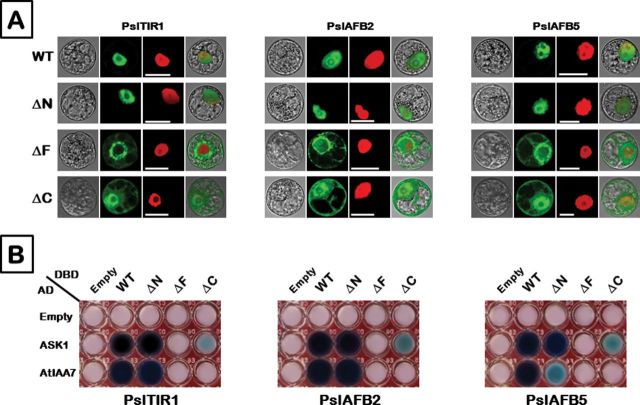
Functional characterization of PslTIR1, PslAFB2, and PslAFB5 domains. Constructs containing full-length ORFs are referred to as WT. Truncated derivatives, including independent deletion of the N-terminal, F-box, and C-terminal domains, are referred to as ∆N, ∆F, and ∆C, respectively. (A) Subcellular localization of WT and truncated derivatives fused to the GFP tag. All constructs were transiently transformed for the assay into *Nicotiana tabacum* protoplasts. *NLS*-mCherry was included in each transfection to indicate the location of the nucleus. GFP fluorescence is shown as green; the merged image is a digital merge of bright field and fluorescent images to illustrate the protein compartments. All experiments were repeated a minimum of three independent times. Bars, 10 µm. (B) Y2H assays were performed using previously indicated derivatives with ASK1 and AtIAA7 in the presence of IAA. Other details as described in Fig. 3.

All *TIR1/AFB* gene family members are structurally conserved across land plant lineages. Their predicted proteins comprise three typical domains—N-terminal, F-box, and C-terminal—that are commonly associated with the F-box subunit protein of SCF (E3) ubiquitin–ligase (Supplementary Figure S2 available at *JXB* online). To explore the structural contribution of each domain in mediating auxin signalling, we performed a series of experiments, including subcellular localization and Y2H assays of full-length ORFs and ORFs containing independent deletions of the three PslTIR1, PslAFB2, and PslAFB5 domains. The full-length proteins are referred to as wild-type (WT). Proteins lacking the N-terminal, F-box, or C-terminal domains are referred to as ∆N, ∆F, or ∆C, respectively. Analysis of TIR1/AFB sequences from different plant species revealed the absence of any feasible targeting sequence that can signify the localization of such protein in the plant cell. Fluorescence microscopy revealed, however, that as expected the full-length PslTIR1/AFBs::GFP fusions were localized exclusively in the nucleus (Fig. 4A). Treatment of cells with IAA before transfection of the GFP fusion construct did not alter the protein localization (data not shown). Deletion of the N-terminal (∆N) domain did not visually affect nuclear localization. By contrast, removal of the C-terminal domain (∆C) noticeably altered the protein localization. Without the C-terminal domain, the GFP signal was uniformly spread throughout the cytoplasm and nucleus. An even more dramatic disruption of protein localization occurred when the F-box domain was eliminated (∆F). Small amounts of the protein localized in the nucleus or cytoplasm; however, most of the GFP signal was observed on the outer surface of the nuclear envelope. To further assess the contribution of each domain in forming SCF-like complexes and activating auxin signalling, the different ORFs were challenged to interact with ASK1 and AtIAA7 in the presence of auxin using a Y2H approach (Fig. 4B). Compared with full-length ORFs, the lack of an N-terminal domain did not noticeably affect the interaction of ∆N with ASK1. Interestingly, although PslTIR1∆N and PslAFB2∆N were able to assemble a co-receptor complex with AtIAA7, the interaction activity of PslAFB5∆N was visibly reduced. The ∆C-derivative remained able to interact with ASK1, albeit at a significantly lower efficiency. In contrast, lack of a C-terminal domain resulted in a complete failure to interact with AtIAA7. Deletion of the F-box domain completely abolished the ability of the protein to interact with ASK1 and AtIAA7. Therefore, although the F-box domain appears to be the key factor in successful Skp1-like protein complex formation, the loss of the C-terminal domain is similarly critical for proper function.

### PslTIR1/AFB expression during fruit development

To investigate the contribution of the plum auxin receptors in fruit development, the accumulation profiles of the three genes were determined at different developmental stages in EG and V9 fruit. During the immature stages (flowering to S3), both cultivars exhibited similar transcript accumulation profiles for all three genes, with slightly, but consistently higher levels, in EG compared with V9 (data not shown).

**Fig. 5.**
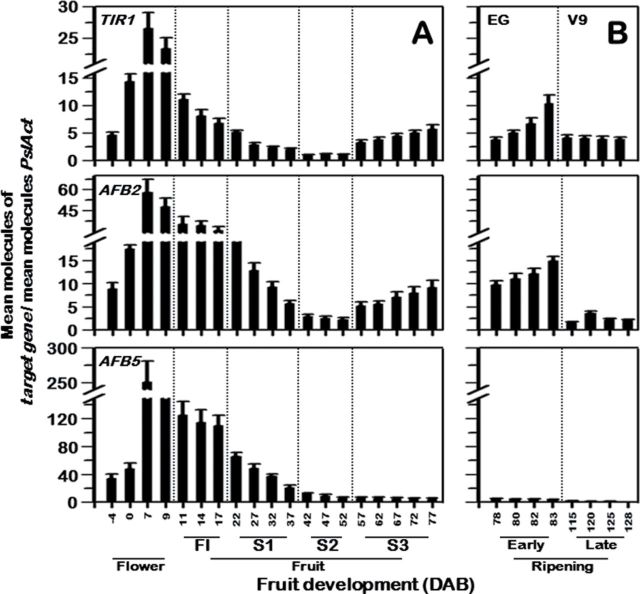
Steady-state transcript levels of *PslTIR1*, *PslAFB2*, and *PslAFB5* mRNAs assessed by qPCR during (A) EG flower and fruit development and (B) throughout ripening of early –EG and late –V9 fruit. The expression was determined from fruit pulp. Results represent data from three biological and three technical replicates. Standard curves were used to calculate the number of target gene molecules per sample. These were then normalized relative to *PslAct* expression. Error bars represent SD. The y axis refers to the mean molecules of the target gene per reaction/mean molecules of PslAct. The x axis in each figure represents the developmental stage as indicated by the number of days after bloom (DAB).

All transcripts were initially present at low levels in flower buds, but accumulated during flower development, peaking soon after fertilization at ~7 DAB (Fig. 5A). The levels detected at this stage were the highest detected over the experiment, with the transcript levels for all three genes declining to very low levels by the S2 stage (~52 DAB). *PslTIR1* and *PslAFB2* transcript levels, however, increased markedly during S3, with the progression to maturity. By contrast, those of *PslAFB5* were barely detectable during this phase.

Transcript levels for the three genes were closely assessed during the S4 stage, where most ripening-related metabolic changes take place. Clear differences in transcript accumulation profiles were observed during this stage (Fig. 5B). In EG, *PslTIR1* and *PslAFB2* levels were low before the onset of the climacteric phase (~78 DAB), but steadily increased with the progression in ripening. In contrast, *PslTIR1* and *PslAFB2* transcript levels during ripening of V9 (~110–128 DAB) remained constitutively low throughout the process. *PslAFB5* transcripts were undetectable during ripening in both cultivars.

### Identification of *PslTIR1/AFB* alleles among plum cultivars

**Fig. 6.**
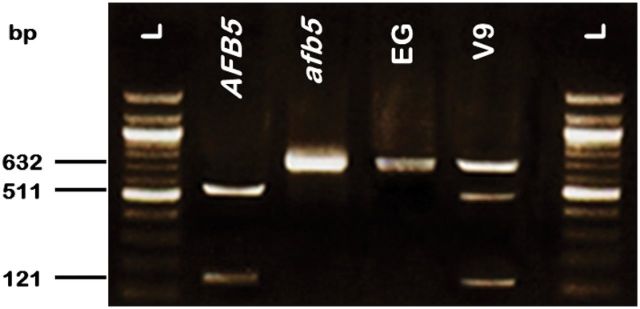
CAPS analysis of PslAFB5 alleles. The PCR products of PslAFB5 exon-1 were amplified using EG and V9 gDNA, digested with AccIII and then electrophoresed on 2.5% agarose gel. Lane L refers to 100-bp ladder; lanes indicated by AFB5 and afb5 are the digested products from subcloned exon-1 fragments. Lanes EG and V9 refer to digested PCR products using the gDNA of the plum cultivars indicated as a template.

In an attempt to further unravel the effects of auxin during fruit development and links between auxin receptors and the variation in auxin sensitivity among cultivars, the full-length genomic sequences of the three genes were isolated and sequenced from EG and V9. All three *gPslTIR1*/*AFB* genes in both cultivars have open reading frames comprising three exons and two introns (Supplementary Figure S4 available at *JXB* online) with the exon/intron boundaries occurring in the same positions in both cultivars (Supplementary Figure S2 available at *JXB* online). The two cultivars were homozygous for *gPslTIR1* and *gPslAFB2*; however, two different alleles were identified for *gPslAFB5*, with the alterations in nucleotide composition leading to four predicted changes in amino acid residues (Supplementary Figure S5 available at *JXB* online). One alteration in particular occurred within the F-box domain, causing a predicted substitution in the highly conserved residue Pro_61_ (Pro to Ser). Pro_61_ has been shown to be essential for TIR1-like function (Gray *et al.*, 1999; Kepinski and Leyser, 2005). Given the nature of the substitution, we predicted that the allelic form with Pro_61_ (designated *gPslAFB5*) would be functional and the allele with the substitution Ser_61_ (designated *gPslafb5*) would be inactive, or at best differentially functional. Strikingly, the auxin-hypersensitive cultivar, EG, was homozygous for *gPslafb5*; and the reduced auxin-sensitivity cultivar, V9, had the *gPslAFB5*/*afb5* heterozygous genotype (Supplementary Figure S6 available at *JXB* online). To confirm the distribution of *gPslAFB5* alleles among cultivars, we performed a cleaved-amplified polymorphic sequence (CAPS) analysis (Fig. 6). Sequence analysis revealed that the exon-1 fragment of *PslAFB5* (632bp) is interrupted by a unique AccIII-restriction site that divides it into two smaller fragments (121 and 511bp). Due to a nucleotide substitution, the AccIII-site is not present in the *gPslafb5* allele. Direct digestion of the amplified *gPslAFB5* exon-1 from the two cultivars confirmed that *gPslAFB5* was represented by a single allele (*gPslafb5*) in EG and by two different alleles in V9.

### The Pslafb5 protein is an inactive auxin-receptor

**Fig. 7.**
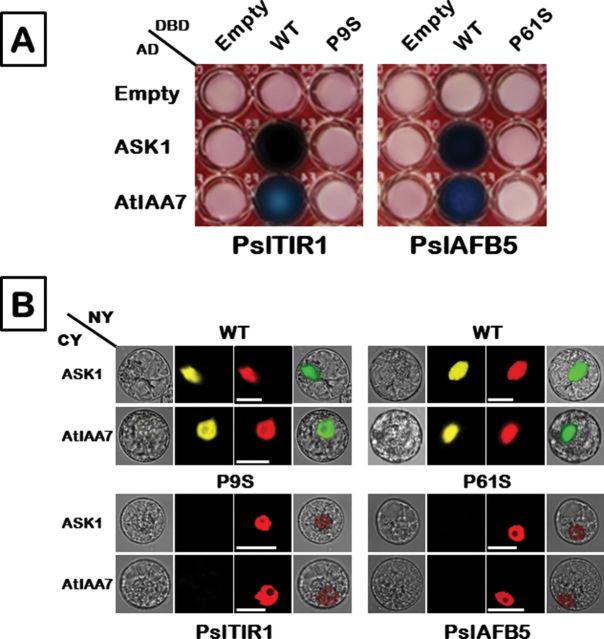
Pslafb5 encodes an inactive auxin receptor. Constructs containing full-length ORFs of PslTIR1 and PslAFB5 were used as a positive control and referred to as WT. Mutations were created by a site-directed mutagenesis approach through substitution of Pro9 and Pro61 of PslTIR1 and PslAFB5, respectively, into serine to simulate Pslafb5. (A) Y2H interaction assays of PslTIR1 and PslAFB5 (WT) and mutated derivatives with ASK1 and AtIAA7 (other details as in Fig. 4). (B) BiFC interaction visualization of PslTIR1 and PslAFB5 (WT and mutated ORFs) with ASK1 and AtIAA7. PslTIR1- and PslAFB5-related proteins were fused with the N-terminus (NY) of YFP; ASK1 and AtIAA7 were fused with the C-terminus (CY) of YFP. Different combinations of NY and CY constructs were transiently co-expressed in tobacco protoplasts. NLS-mCherry was included in each transfection to highlight the location of the nucleus. YFP fluorescence is yellow; the merged image is a digital merge of bright field and fluorescent images to illustrate the interaction location. Bars, 10 µm.

To determine whether the observed Pro_61_-to-Ser mutation in *Pslafb5* influences SCF assembly and/or subsequent co-receptor formation with Aux/IAAs, the corresponding proline residues in PslTIR1 (Pro_9_) and PslAFB5 (Pro_61_) were mutated into serine. The consequences of these mutations were evaluated by assessing the interaction capacity of the full-length WT ORFs (as a positive control) and ORFs carrying mutations, using both Y2H and BiFC approaches (Fig. 7). Relative to WT ORFs, all mutated proteins were unable to interact with ASK1 or AtIAA7 in yeast or tobacco protoplasts, providing strong evidence that the *Pslafb5* allele encodes an inactive TIR1-like protein.

## Discussion

Fruit growth is a complex process where cell division, expansion and differentiation are coordinated by a diverse range of hormones. Auxin is a regulator of patterning and tissue specification in many processes in plants, including the development of reproductive and fruiting tissues (Sundberg and Østergaard, 2009). In flowers, auxin specifies the site of floral primordium initiation and regulates floral organogenesis and patterning (Reinhardt *et al.*, 2000; Cheng and Zhao, 2007). Post-fertilization, an interaction between auxin and gibberellin triggers fruit-set, the term given to the onset of rapid cell division necessary for early embryo development and fruiting structure enlargement (de Jong *et al.*, 2009*a*; El-Sharkawy *et al.*, 2014). In both berry- and stone-fruit, auxin has also been shown to be important during the immature fruit development and ripening processes (Miller *et al.*, 1987; Agustí *et al.*, 1999; Jones *et al.*, 2002; El-Sharkawy *et al.*, 2010, 2012; Seymour *et al.*, 2013). Our results show that the IAA content in plums is high during flowering and in most phases of fruit development. The major exception is the S2 phase of fruit growth where the fruit appears to fall into a quiescent phase. Whereas auxin was once thought to be exclusively involved in the early stages of fruit development (S1 and S3), it is now understood also to be important during the S4 phase, where autocatalytic ethylene production is critical for the suite of changes that comprise the ripening process (Miller *et al.*, 1987; Trainotti *et al.*, 2007). We have previously shown the effect of auxin in enhancing ethylene production and ripening in Shiro, a late plum cultivar (El-Sharkawy *et al.*, 2008, 2009, 2010, 2012). This finding led us to hypothesize that the differences in ripening-associated ethylene production observed in different plum cultivars are at least partially due to variations in the auxin-content and/or -responsiveness in fruiting tissues. The early ripening cultivar, EG, exhibited a sharp increase in auxin levels during fruit ripening and a strong auxin-sensitivity. Treatment with auxin elicited an enhanced ethylene climacteric comparable to that observed in fruit treated with propylene, a known ethylene inducer (McMurchie *et al.*, 1972). Together, the data indicate that auxin plays a key role in progressing fruit development towards the transition phase that leads to the initiation of autocatalytic ethylene production. It also indicates that auxin regulates ethylene production and the progression of ripening. A capacity to advance and enhance ripening in EG suggests that the relative lack of auxin and/or reduced auxin-sensitivity in V9 is, at least partially, responsible for the suppressed ethylene production and prolonged ripening in this cultivar.

To investigate the molecular basis of auxin action in fruit development, three auxin-receptors, belonging to the F-box *TIR1*-like gene family, were isolated and characterized. TIR1-like proteins are components of an SCF–ubiquitin ligase complex that targets the Aux/IAA auxin-repressors for ubiquitination and subsequent degradation (Gray *et al.*, 2001; Dharmasiri *et al.*, 2003). Consistent with published data on TIR1-like proteins, we demonstrated that the isolated PslTIR1/AFBs were able to interact with the receptor complex component, ASK1 and that they were differentially responsive to various Aux/IAA proteins. Interestingly, the absence of auxin did not prevent the assembly of PslTIR1/PslAFB2::AtIAA7; however, the tomato Aux/IAA (SlIAA3) interacts poorly with PslAFB5, even in the presence of auxin. Auxin orchestrates a broad range of processes and many levels of regulation are known to be involved in determining responses (Ljung, 2013), including biosynthesis/metabolism, transport, and response. Our data and those of others (Parry *et al.*, 2009; Calderón Villalobos *et al*., 2012) show that another level of regulation exists at the level of receptor:: auxin:: Aux/IAA-binding capacity.

Assessing the role of different PslTIR1/AFBs domains in protein function confirmed the essential contribution of the F-box and C-terminal domains in ensuring appropriate receptor activity. Loss of the N-terminal domain in PsITIR1 and PslAFB2 had little effect on ligand–receptor complex formation and function; however, the long N-terminal region of PslAFB5 was required for full protein function. Similarly, other studies have also shown the importance of sequences outside the F-box for Skp1 association (Li and Johnston, 1997; Gray *et al.*, 1999).

An analysis of transcripts for three *PslTIR1*/*AFB* mRNAs during EG and V9 fruit development did not correlate with the observed cultivar differences in terms of auxin-sensitivity and fruit development. Sequence data indicated, however, that while only one type of allele was present for two of the receptor genes, *gPslTIR1* and *gPslAFB2*, two different allele types were identified for *gPslAFB5*. The auxin-hypersensitive cultivar, EG, is homozygous for the *gPslafb5* allele, whereas V9 is *gPslAFB5*/*afb5* heterozygous. In the *gPslafb5* allele, a serine residue is substituted for the highly conserved Pro_61_ located within the F-box domain. A targeted mutation in the corresponding proline (Pro_10_ to Ala) of the *Arabidopsis TIR1* abolished auxin responses by inhibiting its capacity to interact with Skp1-like and Aux/IAA proteins (Gray *et al.*, 1999; Kepinski and Leyser, 2005). This suggests that the *Pslafb5* allele encodes an inactive auxin-receptor. In *Arabidopsis*, the loss of the closest homologues of *PslAFB5*, *AFB4*, and/or *AFB5* resulted in a range of growth defects consistent with auxin hypersensitivity, suggesting that this class of auxin receptors act as negative regulators of auxin responses (Greenham *et al.*, 2011). EG is therefore associated with the homozygous presence of the inactive *Pslafb5* allele, and conversely, V9 is associated with the presence of a functional *PslAFB5* allele. Further, we were able to show that a substitution of a highly conserved F-box proline residue (*PslTIR1*–Pro_9_ and *PslAFB5*–Pro_61_) into serine, simulating the naturally occurring sequence in the *Pslafb5* allele, completely abolished auxin responses by inhibiting interactions with ASK1 and AtIAA7. This provides strong evidence that this proline residue is essential for protein function and therefore that the *Pslafb5* allele in the EG cultivar is non-functional.

In the auxin-hypersensitive *Arabidopsis afb4-2* mutant, IAA levels are not significantly altered compared to the wild type (Greenham *et al.*, 2011). Intriguingly, EG also exhibited auxin hypersensitivity. Taken together, the results suggest that the active *PslAFB5* is involved in the auxin-signalling network that negatively regulates fruit development processes, particularly from flowering until the end of the S1 stage. Thus, the loss of *PslAFB5* function may increase auxin responses and consequently accelerate early fruit development processes in EG. Auxin can also be a part of the mechanisms that control the capacity of the fruit to ripen. However, the absence of *PslAFB5* transcripts during late fruit growth and ripening phases (S3 and S4) in both plum cultivars suggested that the auxin content character should be more important than the auxin hypersensitivity feature for determining the auxin-dependence of fruit development and ripening. Auxin acts upstream of ethylene to control the initiation of autocatalytic ethylene production and the transition of a fruit from the end of growth into the onset of ripening. So the high auxin levels in EG should accelerate fruit growth and force the fruit to early transit into ripening; however, the lack of sufficient auxin in V9 delays both processes. Once autocatalytic ethylene production is initiated during ripening, the process will progress in both auxin- and ethylene-dependent manners (El-Sharkawy *et al.*, 2008,^2009^; Ziliotto *et al.*, 2008).

The data presented here provide further evidence of the integral role played by auxin in fruit development, and provide a basis for continuing research to determine the precise nature of the many complex regulatory processes involved.

## Supplementary material

Supplementary data can be found at *JXB* online.

Supplementary Table S1. Oligonucleotide primers used in this study.

Supplementary Table S2. Flowering, maturation dates and fruiting duration in different plum genotypes.

Supplementary Table S3. Changes in plum flower developmental characteristics due to auxin treatment.

Supplementary Table S4. Amino acid sequence comparison between the predicted full-length plum and *Arabidopsis* auxin receptors.

Supplementary Figure S1. Alteration in ethylene production during the ripening of fruits from early and late cultivars pre-exposed to different treatments designed to alter auxin- or ethylene-responses.

Supplementary Figure S2. Amino acid sequence alignment of plum and *Arabidopsis* TIR1/AFB proteins.

Supplementary Figure S3. Phylogenetic relationships between *TIR1/AFB* genes from plum and other plant species.

Supplementary Figure S4. Genomic structure of *PslTIR1/AFB* genes.

Supplementary Figure S5. Amino acid sequence comparison of the two plum *AFB5* alleles (*PslAFB5* and *Pslafb5*).

Supplementary Figure S6. Sequencing traces of *PslAFB5* allelic genotypes in EG and V9 plum cultivars.

## Funding

Partial funding for this work was provided by Ontario Tender Fruit Marketing Board and Ontario Ministry of Agriculture, Food and Rural Affairs to SJ.

## Supplementary Material

Supplementary Data

## Abbreviations:

aa: amino acids
BiFC: bimolecular fluorescence complementation
DAB: days after bloom
EG: Early Golden
S1–4: stage 1–4
V9: V98041 plum genotype
WT: wild-type
Y2H: yeast two-hybrid.
