# Supplementary material for: TIR1-like auxin-receptors are involved in the regulation of plum fruit development
Source: J Exp Bot. 2014 Jul 4;65(18):5205–15. doi: 10.1093/jxb/eru279 (PMC4157706; doi:10.1093/jxb/eru279)
Supplement: Supplementary Data [file supp_65_18_5205__index.html]

TIR1-like auxin-receptors are involved in the regulation of plum fruit development — TIR1-like auxin-receptors are involved in the regulation of plum fruit development — Supplementary Data 

# TIR1-like auxin-receptors are involved in the regulation of plum fruit development

## Supplementary Data

Data files

**Files in this Data Supplement:**

- Supplementary Data - Supplementary Data
