# Supplementary material for: TIR1-like auxin-receptors are involved in the regulation of plum fruit development
Source: J Exp Bot. 2014 Jul 4;65(18):5205–15. doi: 10.1093/jxb/eru279 (PMC4157706; doi:10.1093/jxb/eru279)
Supplement: Supplementary Data [file supp_eru279_jexbot123190_file001.pdf]

Supplemental Information

TIR1-like auxin-receptors are involved on the regulation of plum fruit development.

El-Sharkawy I, Sherif S, Jones B, Mila I, Kumar P, Bouzayen M, Jayasankar S*.

This PDF file includes:

- Supplemental Materials and methods.
- Supplemental Results.
- Supplemental References.
- Supplemental Tables.
- Supplemental Figure Legends.
- Supplemental Figures.

Supplemental Materials and methods

Hormones quantification

Ethylene production was determined during different developmental stages (flowers and fruits) or in treated fruit using gas chromatography. Fruit tissues from five fruits exhibiting similar ethylene production were collected, frozen in liquid-N_2 and stored at –80°C for further analysis. Auxin extraction and quantification were as described previously (Seo *et al*., 2011) using UPLC-ESI-MS/MS system. All experiments were carried out in nine independent biological replicates (5 fruits / replicate).

Isolation and in silico analysis of PslTIR1/AFB *sequences*

Based on the sequence similarity among various *TIR1*-like *F*-box auxin-receptors from different plant species, a pair of degenerate primer (# 1 & 2, Table S1) was designed from the conserved regions to amplify the plum orthologues. The isolated fragments were cloned, sequenced and analyzed using BLAST (Altschul *et al.*, 1997). Extension of the partial cDNA clones were carried out using the 3'- and 5'- RACE kit (Invitrogen, Burlington, ON, Canada). Full-length amplification of cDNA sequences designated *PslTIR1*, *PslAFB2* and *PslAFB5* was carried out using Platinum Taq DNA Polymerase High Fidelity, following the instructions provided by the manufacturer (Invitrogen). Alignment of predicted proteins was performed using Clustal*X* and the neighbor-joining tree was generated with MEGA5 (Tamura *et al.*, 2011). Structural domains were annotated using online software 'Smart' (Letunic *et al.*, 2009). Full-length genomic sequences of

gPslTIR1/AFBs were isolated from different plum cultivars using the AccuPrime *Pfx* (Invitrogen).

DNA, RNA extraction and qPCR assays

Genomic DNA was extracted from young plum leaves according to the DNeasy Plant Maxi Kit (Qiagen, Mississauga, ON, Canada). Total RNA extraction, DNase treatment, cDNA synthesis and qPCR reactions were performed as described previously (El-Sharkawy *et al.*, 2012b). Gene-specific primers were designed using Primer Express (v3.0, Applied Biosystems, Carlsbad, CA, USA) (primers # 3-10, Table S1). Three technical replicates from three biological replicates for each reaction were analyzed on an ABI PRISM 7900HT Sequence Detection System (Applied Biosystems). Transcript abundance was quantified using standard curves for both target and reference genes [*PslAct* (EF585293)], which were generated from serial dilutions of PCR products from corresponding cDNAs.

Protoplast isolation and transient expression of PslTIR1-, PslAFB2-, *and* PslAFB5-*GFP fusion proteins*

Full-length and modified *PslTIR1*, *PslAFB2* and *PslAFB5* ORFs were fused in frame with the GFP into the pGreenII vector using the *Bam*HI site and expressed under the control of *35S* promoter. Protoplasts used for transfection were obtained from suspension-cultured tobacco *BY-2* cells. Protoplasts were transfected with the constructs in the presence or absence of 100 µM IAA and analyzed for GFP

fluorescence by confocal microscopy as described previously (El-Sharkawy *et al.*, 2009). All assays were repeated at least five times.

Internal deletion of PslTIR1*,* PslAFB2 *and* PslAFB5 F-*box domain*

In case of *PslTIR1* and *PslAFB2* that exhibit short *N*-terminal region (21-*bp* and 12-*bp*, respectively); the *C*-terminal fragment was amplified and cloned using gene-specific primer pairs. Then, a long forward primer, which includes the full *N*-terminal region with a tail that contains the beginning of the *C*-terminal fragment was used with applicable reverse primer to amplify the full *PslTIR1ΔF*-box and *PslAFB2ΔF*-box fragments (primers # 23-26, Table S1). However, another strategy was used to generate the truncated *F*-box domain of *PslAFB5* sequence that displays longer *N*-terminal region (177-*bp*) as follow: (i) The *N*-terminal (fragment A) and *C*-terminal (fragment B) were independently amplified (primers # 27-30, Table S1), in which the reverse and forward primers of fragments A and B, respectively, were phosphorylated. (ii) The PCR products were purified and diluted to a final concentration 20 ng/µl. (iii) A ligation reaction was set up using 50 ng of each fragment, (iv) then a PCR reaction was carried out using the forward and reverse primers of the fragments A and B, respectively, in the presence of 2 µl of ligation reaction as a template, which generated the *PslAFB5ΔF*-box fragment.

Accession numbers

Sequence data used in this study can be found in GenBank or EMBL database under the following accession numbers: *PslTIR1* (KJ018741), *gPslTIR1*

(KJ018742), *PslAFB2* (KJ018743), *gPslAFB2* (KJ018744), *PslAFB5* (KJ018745), *gPslAFB5* (KJ018746), *Pslafb5* (KJ018747), *gPslafb5* (KJ018748), *ASK1* (U70034), *AtIAA7* (U18409), *SlIAA3* (JN379433), *SlIAA9* (JN379437).

Supplemental Results

Structural characteristics of auxin receptors PslTIR1/AFBs

The auxin, Aux/IAA and $InsP_6$ substrates binding sites are highly conserved across *PslTIR1/AFBs* with some minor changes. Recently, Yu *et al*. (2013) reported the identification of two mutations (D_{170}-to-E and M_{473}-to-L) that are uniquely effective in TIR1 protein and act either independently or mutually via increasing TIR1-ineteraction strength with Aux/IAA proteins, resulting in auxin-hypersensitivity. Consistent with *AtTIR1*, D_{170} residue is conserved in *PslTIR1*; however, M_{473} residue is substituted to L, which should potentially increase the auxin-hypersensitivity capacity of the protein (Fig. S2).

An un-rooted phylogenetic tree was constructed to assess the relationships between plum sequences and other homolog sequences from different plant species (Fig. S3). Despite plant nature, monocot or dicot, the dendrogram structurally divided the global *TIR1/AFB* sequences of flowering plants into three distinguishable subfamilies, in which plum sequences have a representative in each subfamily. Establishing a correlation between phylogenesis, sequence characteristics and functional properties indicated that predicted *PslTIR1* and *PslAFB2* are classified as members of subfamily-I and -II that comprises *TIR1/AFB1*- and *AFB2/AFB3*-related genes, respectively. Both subfamilies members act as positive regulators of auxin-signaling (Dharmasiri *et al.*, 2005b; Parry *et al.*, 2009) and are structurally close due to holding a short *N*-terminal region that varies in length among the different subfamily-I members (4-8 aa); however, this region constantly consists of three amino acid residues in subfamily-

II. *PslAFB5* predicted protein assigns to subfamily-III that act as negative regulators of auxin-signaling (Greenham *et al.*, 2011) and structurally includes all *AFB* proteins with highly extended *N*-terminal length (30-58 aa). Although the function of this extension is unknown, it is rich in serine residues and motif analysis programs predict several phosphorylation sites, which suggested conserved function.

Supplemental References

Altschul SF, Madden TL, Schaffer AA, Zhang J, Zhang Z, Miller W, Lipman DJ. 1997. Gapped BLAST and PSI-BLAST: a new generation of protein database search programs. *Nucleic Acids Research* **25,** 3389-3402.

El-Sharkawy I, El Kayal W, Prasath D, Fernández H, Bouzayen M, Svircev AM, Jayasankar S. 2012b. Identification and genetic characterization of a gibberellin 2-oxidase gene that controls tree stature and reproductive growth in plum. *Journal of Experimental Botany* **63,** 1225-1239.

Letunic I, Doerks T, Bork P. 2009. SMART 6: recent updates and new developments. *Nucleic Acids Research* **37,** D229-D232.

Seo M, Jikumaru Y, Kamiya Y. 2011. Profiling of hormones and related metabolites in seed dormancy and germination studies. *Methods in Molecular Biology* **773,** 99-111.

Tamura K, Peterson D, Peterson N, Stecher G, Nei M, Kumar S. 2011. MEGA5: Molecular Evolutionary Genetics Analysis using maximum likelihood, evolutionary distance, and maximum parsimony methods. *Molecular Biology and Evolution* **28,** 2731-2739.

Tan X, Calderon-Villalobos LIA, Sharon M, Zheng C, Robinson CV, Estelle M, Zheng N. 2007. Mechanism of auxin perception by the TIR1 ubiquitin ligase. *Nature* **446,** 640-645.

Yu H, Moss B, Jang SS, Prigge M, Klavins E, Nemhauser J, Estelle M. 2013. Mutations in the TIR1 auxin receptor that increase affinity for Aux/IAA proteins result in auxin hypersensitivity. *Plant Physiology* **162,** 295-303.

Zuckerkandl E, Pauling L. 1965. Evolutionary divergence and convergence in proteins. In Evolving Genes and Proteins. Edited by Bryson, V. and Vogel, H.J. pp. 97-166. Academic Press, New York.

Supplemental Tables

Table S1. The oligonucleotide primers.

Name	Oligonucleotide sequence
**Genes isolation**	
**1-PslAFB(F)**	**5'-**ACRCTTCKCCGACTTCAATCTGRTGC**-3'**
**2-PslAFB(R)**	**5'-**GCWGTCCCTGATCTCAAGTTTC**-3'**
**QRT-PCR**	
**3-PslTIR1(FQ)**	**5'**-CTCTCGGGATGCAAGGAA-**3'**
**4-PslTIR1(RQ)**	**5'**-TGATGTCAGCCCAGAGCA-**3'**
**5-PslAFB2(FQ)**	**5'**-GACGCTGTCACCATGCAG-**3'**
**6-PslAFB2(RQ)**	**5'**-CAGTCAAAAGGCCGGAGA-**3'**
**7-PslAFB5(FQ)**	**5'**-TCTCCGCCGTTTGATCTCA-**3'**
**8-PslAFB5(RQ)**	**5'**-CAGTTCCGGGCCTTGTTG-**3'**
**9-PslAct(FQ)**	**5'**-CTGGACCTTGCTGGTCGT-**3'**
**10-PslAct(RQ)**	**5'**-ATTTCCCGCTCAGCAGTG-**3'**
**Generate truncated sequences**	
**11-PslTIR1-ΔN(F)**	**5'**-ATGTTCCCAGAGGAGGTGCTAGAA-**3'**
**12-PslTIR1(R)**	**5'**-AGTAACCCTCACTGCAGAATCTTCATC-**3'**
**13-PslAFB2-ΔN(F)**	**5'**-ATGTTTCCAGACGAGGTAATAGAG-**3'**
**14-PslAFB2(R)**	**5'**-CAGAGTCCACACAAACTCCGGTTT-**3'**
**15-PslAFB5-ΔN(F)**	**5'**-ATGTACCCGGACCAAGTCCTCGAGA-**3'**
**16-PslAFB5(R)**	**5'**-TAGGATCTCAACAAACTTTGAAATAT-**3'**
**17-PslTIR1(F)**	**5'**-ATGCTGAAAATGGCGAACTCGTTC-**3'**
**18-PslTIR1-ΔC(R)**	**5'**-GAATATTCTCCTCCTGCACCAT-**3'**
**19-PslAFB2(F)**	**5'**-ATGAATTACTTTCCAGACGAGGTA-**3'**
**20-PslAFB2-ΔC(R)**	**5'**-TATGAACACTCTCTCCCTACTAAA-**3'**
**21-PslAFB5(F)**	**5'**-ATGGGGGAGGACCCTTCA-**3'**
**22-PslAFB5-ΔC(R)**	**5'**-GATGAAGAGCTCGGATCG-**3'**
**23-PslTIR1-ΔF(F)**	**5'**-ATGCTGAAAATGGCGAACTCGATCGGGAACTGCTAC-**3'**
**24-PslTIR1(R)**	**5'**-AGTAACCCTCACTGCAGAATCTTCATC-**3'**
**25-PslAFB2-ΔF(F)**	**5'**-ATGAATTACGGGAATTGCTATGCGAT-**3'**
**26-PslAFB2(R)**	**5'**-CAGAGTCCACACAAACTCCGGTTT-**3'**
**27-PslAFB5(F)**	**5'**-ATGGGGGAGGACCCTTCA-**3'**
**28-PslAFB5-Nter(Rp)**	**5'**-GGGGACGCTGTACTCGATGGA-**3'**
**29-PslAFB5-Cter(Fp)**	**5'**-GGCAACTGCTATGCGGTCTCT-**3'**
**30-PslAFB5(R)**	**5'**-TAGGATCTCAACAAACTTTGAAATAT-**3'**

Table S2: Flowering, maturation dates and fruiting duration of in different plum genotypes.

Selection	Flowering date	Maturation date	Fruiting duration (d)
Early plum genotypes			
V84041	~ May 04	~ July 18	74
Ouishi-Wase	~ May 05	~ July 23	77
V86041	~ May 03	~ July 25	80
Early Golden	~ April 30	~ July 22	83
V82041	~ May 02	~ July 30	91
Late plum genotypes			
Shiro	~ May 05	~ August 17	103
Obil'naya	~ May 05	~ August 25	111
Vanier	~ May 02	~ August 29	119
Elephant Heart	~ May 06	~ Sept. 03	119
V98041	~ May 09	~ Sept. 09	125

The dates were determined throughout 5 years.

Table S3. Effect of auxin application on EG and V9 flower development process as shown in Fig. 1B.

	**Control**				**NAA 10 μM**				**NAA 100 μM**			
**Flower Stage**	**EG**		**V9**		**EG**		**V9**		**EG**		**V9**	
	**Length**	**Width**	**Length**	**Width**	**Length**	**Width**	**Length**	**Width**	**Length**	**Width**	**Length**	**Width**
**Stage I**	**3.7(±0.8)**	**3.9(±0.1)**	**3.9(±0.6)**	**4.1(±0.3)**	5.9(±0.4)**	4.5(±0.1)*	5.0(±0.5)[NS]	4.6(±0.6)[NS]	3.8(±0.5)[NS]	4.0(±0.1)[NS]	6.5(±0.4)**	5.9(±0.3)**
**Stage II**	**6.1(±0.4)**	**4.5(±0.3)**	**6.3(±0.3)**	**4.9(±0.4)**	9.1(±0.2)**	8.4(±0.3)**	6.2(±0.3)[NS]	5.1(±0.5)[NS]	6.7(±0.5)[NS]	4.6(±0.7)[NS]	8.9(±0.8)**	7.5(±0.4)**
**Stage III**	**7.0(±0.3)**	**5.5(±0.4)**	**7.6(±0.8)**	**6.2(±0.5)**	17.2(±0.8)**	13.0(±0.5)**	8.3(±0.4)[NS]	6.5(±0.6)[NS]	7.6(±0.4)[NS]	6.5(±0.6)[NS]	18.6(±0.5)**	15.0(±0.4)**
**Stage IV**	**17.4(±1.2)**	**15.2(±1.0)**	**17.8(±1.1)**	**16.2(±0.8)**	27.7(±1.2)**	30.8(±0.8)**	18.6(±0.6)[NS]	17.4(±0.9)[NS]	19.5(±1)[NS]	16.9(±1.2)[NS]	27.0(±1.1)**	33.6(±1.2)**

Early (EG) and late (V9) plum cultivars were treated with two different concentrations of NAA, 10 and 100 μM. Floral stages were selected when ~70% of the flowers in the shoot were in the same developmental stage. The stages I, II, III and IV represent flower's age at 2, 3, 4 and 6-days after treatment. The measurements are the means (±SD) of the length and the width (mm) of 24 floral buds (without stalk) or flowers. Measurements were taken in two growing seasons. Statistically significant differences from the control are indicated by (*****) and (******) for the probability levels (P<0.05) and (P<0.01), respectively. NS, non-significant (P>0.05).

Table S4: Amino acid sequence comparison between the predicted full length plum and *Arabidopsis* auxin receptors.

Gene	Protein size (aa)	Amino acid sequence similarity (%)		
		**PslTIR1**	**PslAFB2**	**PslAFB5**
P. salicina				
**PslTIR1**	584	-		
**PslAFB2**	572	74	-	
**PslAFB5**	632	60	60	-
A. thaliana				
**AtTIR1**	594	89	72	57
**AtAFB1**	585	79	70	56
**AtAFB2**	575	73	87	59
**AtAFB3**	577	73	86	60
**AtAFB4**	623	60	61	76
**AtAFB5**	619	60	63	80

Supplemental Figure Legends

Fig. S1. Ethylene production of early (EG) and late (V9) fruit treated with propylene (A, B), 1-MCP (C, D), NAA (E, F) and TIBA (G, H). For auxin treatment EG and V9 fruit treated with NAA at concentrations 10 µM and 100 µM, respectively. Untreated fruit (black circles) were used as controls for each treatment (open circles).

Fig. S2. Amino acid sequence alignment of plum and *Arabidopsis TIR1*/*AFB* proteins using ClustalX program. Conserved residues are shaded in black. Dark grey shading indicates similar residues in seven out of nine of the sequences and clear grey shading indicates similar residues in five out of nine of the sequences. The filled bar below the sequences represents the *F*-box domain. The conserved LRR domains are underlined with thin line. Excluding *PslAFB2*, all sequences shared the absence of LRR3; however, *PslAFB5* is further lacking the LRR4. The green, blue and red circles represent the amino acid residues involved in the binding of auxin, Aux/IAA peptide and InsP6, respectively (Tan *et al.*, 2007). The gray triangles indicate the amino acid residues that control auxin-hypersensitivity in *TIR1*-type proteins (Yu *et al.*, 2013). The yellow triangles indicate the positions of introns occurring among the various *PslTIR1*/*AFBs*.

Fig. S3. Evolutionary relationships of *PslTIR1/AFB* genes. The evolutionary distances were computed using the Poisson correction method (Zuckerkandl and Pauling, 1965). The analysis involved 47 amino acid sequences from different plant species that belong to monocots and dicots. Bootstrap confidence values from 1000 replicates are indicated above branches.

Fig. S4. Genomic structure of *PslTIR1/AFB* genes. The black boxes represent the exons and the grey lines the introns. The white boxes in the *N*-terminal exon represent the position of the putative *F*-box domain. Number of base pairs (bp) refers to full-length genomic sequence from the start codon to the stop codon. Number of amino acid residues (aa) is the full-length predicted protein sequence.

Fig. S5. Amino acid sequence comparison of the two *PslAFB5* alleles. The gray bar and lines under the sequences represent the *F*-box and LRRs domains, respectively. Red highlighted amino acid residue pointed out a silent SNP where changes occurred in nucleotides without alteration in amino acid residue, other details as in Fig. S1.

Fig. S6. Sequencing traces of *PslAFB5* allelic genotypes in EG and V9. The arrows indicate the position where the mutation occurs in the *F*-box domain of *PslAFB5*/*afb5* alleles.

Supplemental Figures

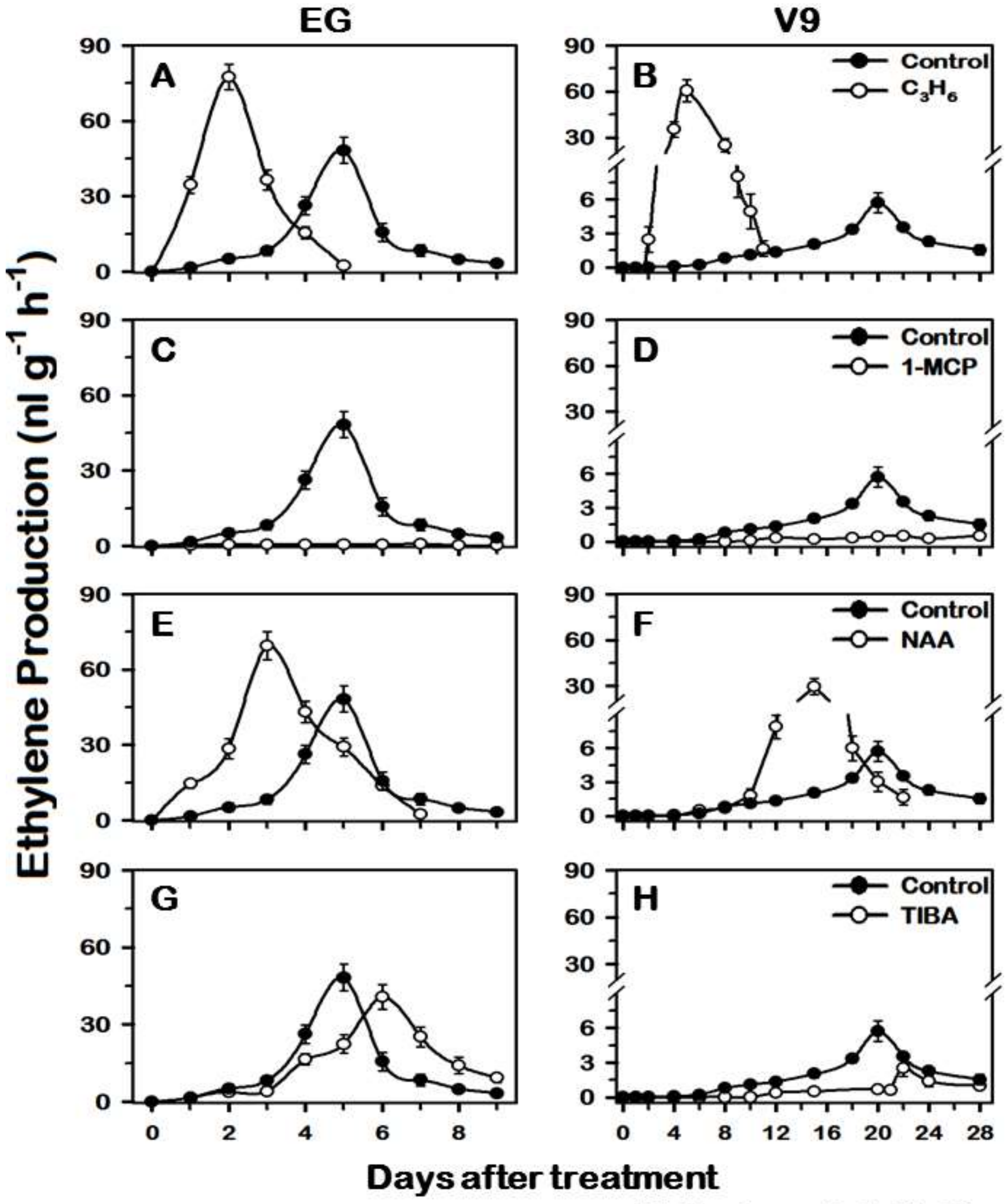

El-Sharkawy et al., Fig.S1

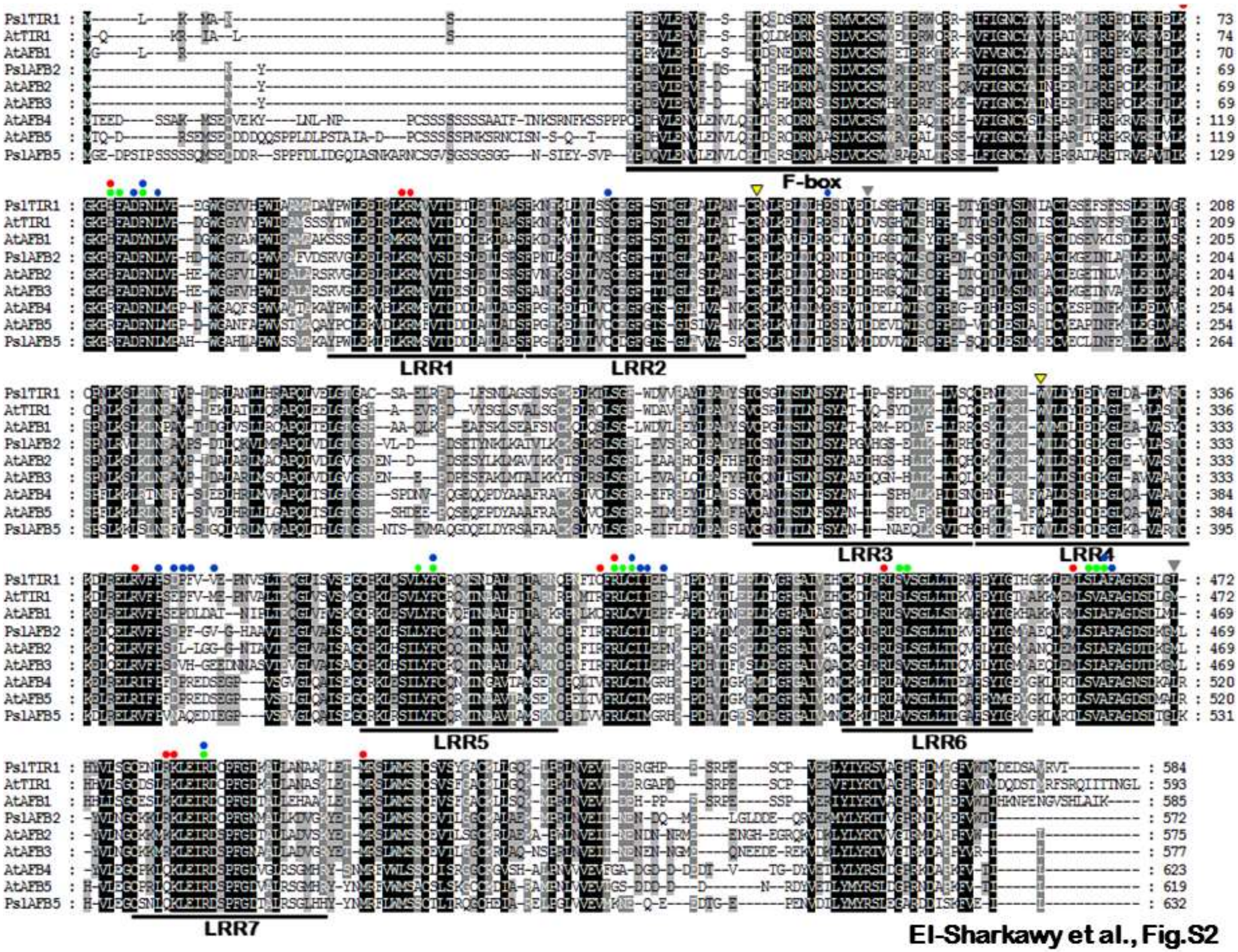

El-Sharkawy et al., Fig.S2

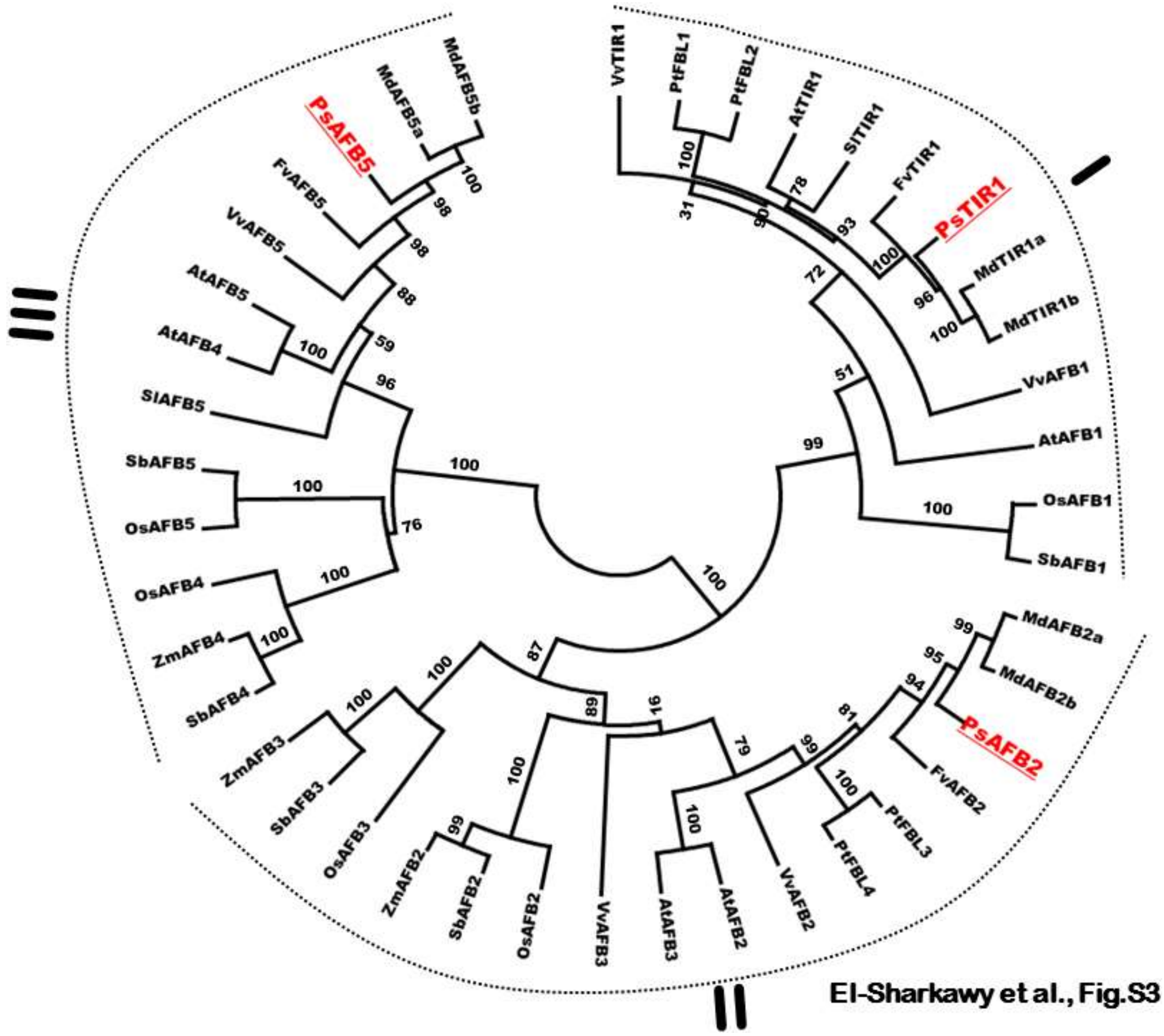

El-Sharkawy et al., Fig.S3

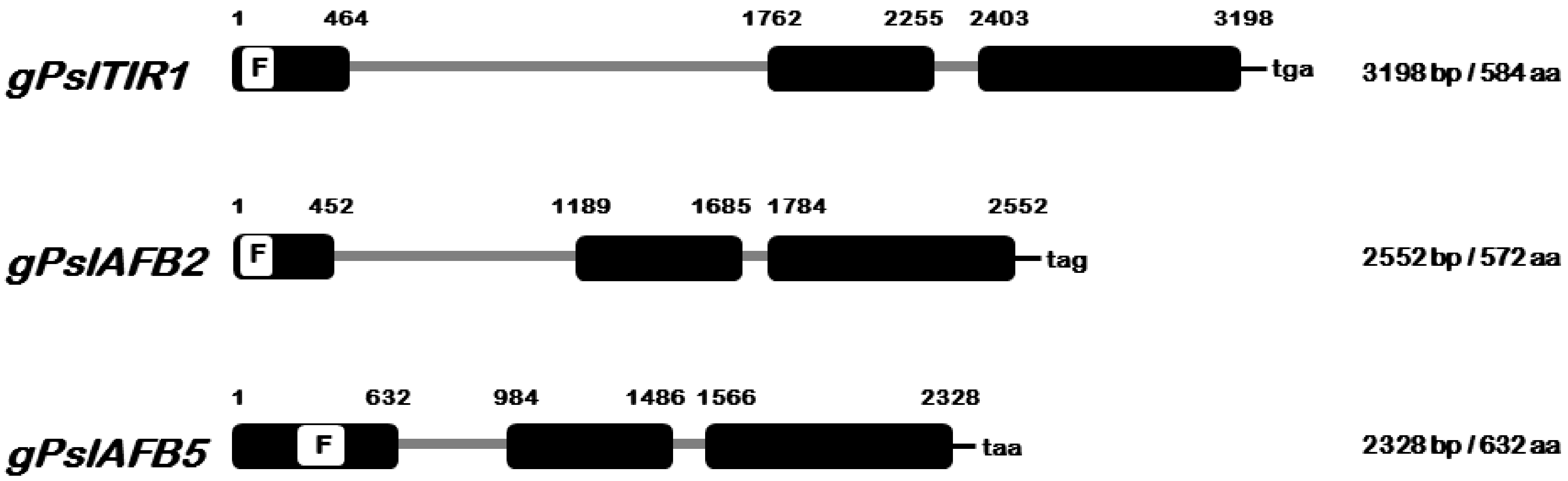

El-Sharkawy et al., Fig. S4

***PsIAFB5* alleles:**

```
                   *        20         *        40         *        60         *        80         *       100         *       120
PslAFB5 : MGEDPSIPSSSSSQMSEDDDRSPPFDLIDGQIASNKARNCSGVSGSSGSGGNSIEYSVPYPDQVLENVLENVLCFLTSRSDRNAASLVCKSWYRAEALTRSELFIGNCYAVSPRRATARF : 120
Pslafb5 : MGEDPSIPSSSSSQMSEDDDRSPPFDLIDGQIASNKARNCPGVSGSSGSGGNSIEYSVPYSDQVLENVLENVLCFLTSRSDRNAASLVCKSWYRAEALTRSELFIGNCYAVSPRRATARF : 120

                   *       140         *       160         *       180         *       200         *       220         *       240
PslAFB5 : TRVRAVTIKGKPRFADFNLMPAHWGAHLAPWVSSMAKAYPWLEKLFLKRMSVTDDDLALLAESFPGFKELVLVCCDGFGTSGLAVVASKCRQLRVLDLTESDVMDDDVDWIRCFPESQTC : 240
Pslafb5 : TRVRAVTIKGKPRFADFNLMPAHWGAHLAPWVSSMAKAYPWLEKLFLKRMSVTDDDLALLAESFPGFKELVLVCCDGFGTSGLAVVASKCRQLRVLDLTESDVMDDDVDWIRCFPESQTC : 240

                   *       260         *       280         *       300         *       320         *       340         *       360
PslAFB5 : LESLMFECVECLINFEALEKLVARSPSLKKLSLNRFVSIGQLYRLMVRAPQLTHLGTGSFNTSEVMAQGDQELDYRSAFAACKSLVYLSGFREIFLDYLPAISPVCGNLTTLNFSYANIN : 360
Pslafb5 : LESLMFECVECLINFEALEKLVARSPSLKKLSLNRFVSIGQLYRLMVRAPQLTHLGTGSFNTSEVMAQGDQELDYRSAFAACKSLVYLSGFREIFLDYLPAISPVCGNLTTLNFSYANIN : 360

                   *       380         *       400         *       420         *       440         *       460         *       480
PslAFB5 : AEQLKSVICHCHKLQTFWVLDSICDEGLKAVARTCKDLRELRVFPVNAQEDIEGPVSEVGLQAISEGCRKLRSILYFCQRMTNAAVIAMSKNCPDLVVFRLCIMGRHRPDHVTGESMDEG : 480
Pslafb5 : AEQLKSVICHCHKLQTFWVLDSICDEGLKAVARTCKDLRELRVFPVNAQEDIEGPVSEVGLQAISEGCRKLRSILYFCQRMTNAAVIAMSKNCPDLVVFRLCIMGRHRPDHVTGESMDEG : 480

                   *       500         *       520         *       540         *       560         *       580         *       600
PslAFB5 : FGAIVMNCKKLTRLAVSGLLTDGAFSYIGKYGKLVRTLSVAFAGDSDTGLKHVLEGCSNLQKLEIRDSPFGDTALRSGLHHYYNMRFLWMSSCTLTRHGCHEIARELPGLVVEVMKNEQE : 600
Pslafb5 : FGAIVMNCKKLTRLAVSGLLTDGAFSYIGKYGKLVRTLSVAFAGDSDTGLKHVLEGCSNLQKLEIRDSPFGDTALRSGLHHYYNMRFLWMSSCTLTRQGCHEIARELPGLVVEVMKNEQE : 600

                   *       620         *
PslAFB5 : KNEQEEDTGEPENVDILYMYRSLEGARDDISKFVEIL : 632
Pslafb5 : KNEQEEDTGEPENVDILYVYRSLEGARDDISKFVEIL : 632
```

El-Sharkawy et al., Fig. S5

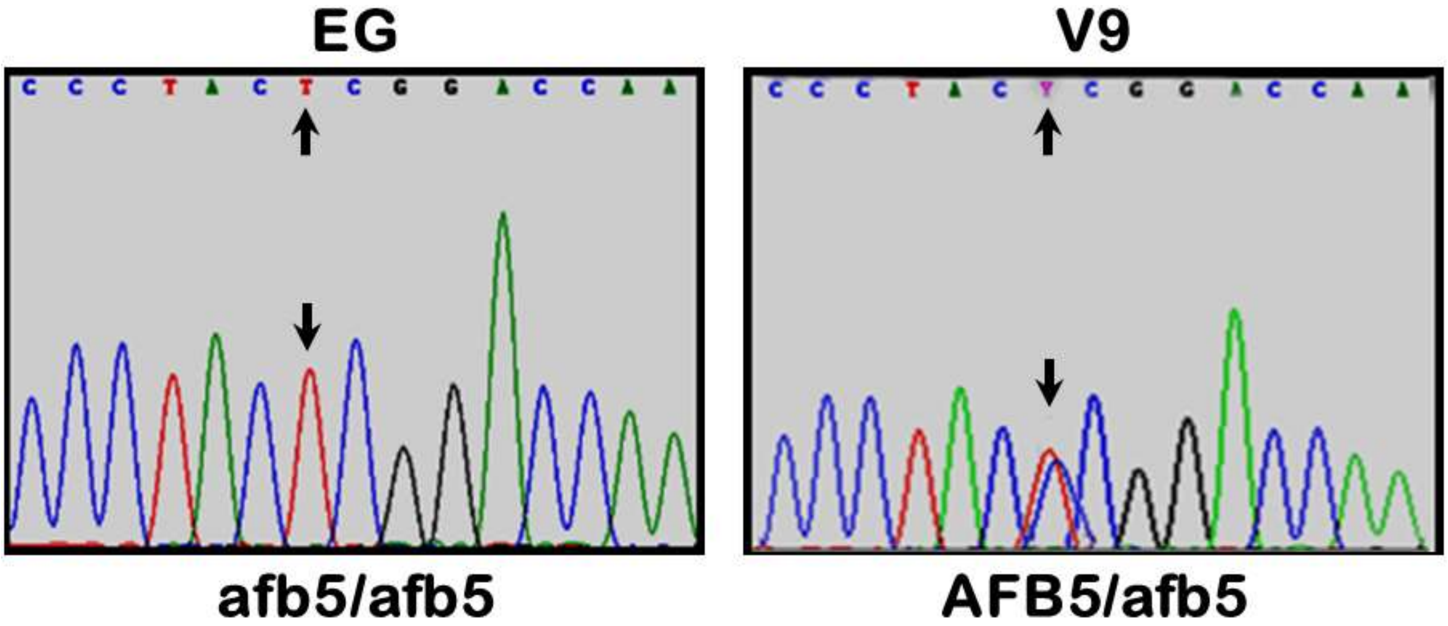

El-Sharkawy et al., Fig. S6
